# Supplementary material for: Dragon boat exercise reshapes the temporal-spatial dynamics of the brain
Source: PeerJ. 2024 Jun 28;12:e17623. doi: 10.7717/peerj.17623 (PMC11216202; doi:10.7717/peerj.17623)
Supplement: Supplemental Information 3 [file peerj-12-17623-s003.docx]

Mann-Whitney U test for microstate transition results

| Time | Group | Transition Probability | Z | p |
| --- | --- | --- | --- | --- |
| Pre | Amateur vs. Professional | A→D  0.072(0.039~0.092) | -2.045 | 0.016 |
|  |  | 0.088(0.071~0.116) |  |  |
|  |  | C→D |  |  |
|  |  | 0.065(0.045~0.081) | -2.043 | 0.041 |
|  |  | 0.073(0.044~0.127) |  |  |
| Post | Amateur vs. Professional | B→D |  |  |
|  |  | 0.058(0.037~0.071) | -2.046 | 0.042 |
|  |  | 0.085(0.045~0.129) |  |  |
|  |  | C→D |  |  |
|  |  | 0.079(0.036~0.131) | -2.431 | 0.019 |
|  |  | 0.107(0.076~0.158) |  |  |
|  |  | D→C |  |  |
|  |  | 0.076(0.035~0.123) | -2.041 | 0.041 |
|  |  | 0.109(0.072~0.159) |  |  |

Wilcoxon test for microstate transition results

| Group | Time | Transition Probability | Z | p |
| --- | --- | --- | --- | --- |
| Amateur | Pre vs. Post | A→B  0.083(0.054~0.119) | -2.159 | 0.031 |
|  |  | 0.051(0.026~0.076) |  |  |
|  |  | B→C |  |  |
|  |  | 0.072(0.039~0.092) | -2.190 | 0.029 |
|  |  | 0.101(0.059~0.136) |  |  |
|  |  | B→D |  |  |
|  |  | 0.086(0.052~0.134) | -2.007 | 0.045 |
|  |  | 0.058(0.037~0.077) |  |  |
| Professional | Pre vs. Post | D→C |  |  |
|  |  | 0.109(0.072~0.159) | -2.029 | 0.043 |
|  |  | 0.072(0.044~0.127) |  |  |
|  |  | B→C |  |  |
|  |  | 0.070(0.519~0.081) | -2.229 | 0.026 |
|  |  | 0.081(0.063~0.120) |  |  |
